# Elucidating the mechanisms of formononetin in modulating atherosclerotic plaque formation in ApoE-/- mice

**DOI:** 10.1186/s12872-024-03774-6

**Published:** 2024-02-22

**Authors:** Ying He, Youde Cai, Dingling Wei, Liping Cao, Qiansong He, Yazhou Zhang

**Affiliations:** 1 First Clinical Medical College, Guizhou University of Traditional Chinese Medicine, Guiyang, Guizhou 550001 China; 2 Jinyang Hospital Affiliated to Guizhou Medical University, Guiyang, Guizhou 550081 China; 3 College of Pharmacy, Guizhou University of Traditional Chinese Medicine, Guiyang, Guizhou 550025 China

**Keywords:** Formononetin, α7nAChR, Atherosclerosis, JAK/STAT, Macrophages

## Abstract

**Background:**

Atherosclerosis(AS) poses a pressing challenge in contemporary medicine. Formononetin (FMN) plays a crucial role in its prevention and treatment. However, the detailed impact of FMN on the stability of atherosclerotic plaques and its underlying mechanisms remain to be elucidated.

**Methods:**

An intervention consisting of FMN was given along with a high-fat food regimen in the ApoE-/- mouse model. The investigation included the evaluation of the degree of atherosclerotic lesion, the main components of the plaque, lipid profiles, particular markers indicating M1/M2 macrophage phenotypes, the quantities of factors related to inflammation, the infiltration of macrophages, and the identification of markers linked to the α7nAChR/JAK2/STAT3 axis effect molecules.

**Results:**

The evaluation of aortic morphology in ApoE-/-mice revealed that FMN significantly improved the plaque area, fibrous cap protrusion, lipid deposition, and structural alterations on the aortic surface, among other markers of atherosclerosis,and there is concentration dependence. Furthermore, the lipid content of mouse serum was assessed, and the results showed that the low-, medium-, and high-dosage FMN groups had significantly lower levels of LDL-C, ox-LDL, TC, and TG. The results of immunohistochemical staining indicated that the low-, medium-, and high-dose FMN therapy groups had enhanced CD206 expression and decreased expression of CD68 and iNOS. According to RT-qPCR data, FMN intervention has the potential to suppress the expression of iNOS, COX-2, miR-155-5p, IL-6, and IL-1β mRNA, while promoting the expression of IL-10, SHIP1, and Arg-1 mRNA levels. However, the degree of inhibition varied among dosage groups. Western blot investigation of JAK/STAT signaling pathway proteins and cholinergic α7nAChR protein showed that p-JAK2 and p-STAT3 protein expression was suppressed at all dosages, whereas α7nAChR protein expression was enhanced.

**Conclusions:**

According to the aforementioned findings, FMN can reduce inflammation and atherosclerosis by influencing macrophage polarization, blocking the JAK/STAT signaling pathway, and increasing α7nAChR expression.

**Supplementary Information:**

The online version contains supplementary material available at 10.1186/s12872-024-03774-6.

## Background

Atherosclerosis is a widely prevalent chronic cardiovascular disease characterized primarily by lipid deposition and chronic inflammatory responses within arterial walls. This condition leads to the formation of plaques, which can accumulate within the arteries, ultimately posing a threat to the normal functioning of the entire cardiovascular system [1]. With the continuous rise in cardiovascular diseases and their associated high disability and mortality rates, atherosclerosis has emerged as an urgent challenge in contemporary medical science [2, 3]. Macro phages play a pivotal role in the pathological process of atherosclerosis. They can polarize into two major subtypes, namely, M1 and M2 [4]. M1 macrophages typically exhibit proinflammatory characteristics, releasing proinflammatory cytokines such as tumor necrosis factor-alpha (TNF-α) and interleukin-1 beta (IL-1β). In contrast, M2 macrophages display anti-inflammatory properties, secreting cytokines such as Fizz1, Arg-1, Ym1, and IL-10 [5, 6]. Maintaining the balance between M1 and M2 macrophages is crucial for regulating the inflammatory response [7]. Cytokines in the inflammatory response play a crucial role in arterial wall inflammation and may contribute to plaque instability. Therefore, modulating the inflammatory response is of paramount importance in the treatment of atherosclerosis [8].

The JAK/STAT signaling pathway is a signaling pathway associated with inflammation [9]. Numerous studies have demonstrated its ability to enhance and prolong the proinflammatory phenotype of macrophages, leading to the secretion of a significant amount of proinflammatory cytokines, such as IL-6 and vascular cell adhesion molecule-1, thereby exacerbating atherosclerosis [10, 11]. Additionally, in the early twentieth century, researchers recognized the importance of neural mechanisms in regulating inflammation, particularly the vagus nerve, and the anti-inflammatory signaling of the vagus nerve is mediated by α7nAChR (α7 nicotinic acetylcholine receptor), meanwhile, α7nAChR is an important downstream effector of the JAK2/STAT3 signaling pathway [12]. These mechanisms can mitigate the inflammatory response within atherosclerotic plaques, simultaneously reducing blood pressure and lipid levels, closely associated with the development of atherosclerosis [13, 14].

In the field of atherosclerosis prevention and treatment, traditional Chinese medicine has played an exceptionally significant role, given its minimal side effects and remarkable efficacy. Several active components have been isolated from various Chinese medicinal herbs, including Chai hu Soap-Saponin A, Ku Shen G, Mu Xi Cao Su, and β-Olive. Research has already established their positive effects in the prevention and treatment of atherosclerosis [15, 16]. Formononetin (FMN) [IUPAC:7-hydroxy-3-(4-methoxyphenyl) chromene 4- one] is one of the primary flavonoid components extracted from plants [17]. In recent years, extensive research has been conducted on the pharmacological actions of FMN, revealing its roles not only in antitumor and neuroprotection but also in protecting the cardiovascular system and diabetic cardiomyopathy. Some studies suggest that FMN plays a significant role in the prevention and treatment of atherosclerosis [18, 19]. However, the impact of FMN on the stability of atherosclerosis and its underlying mechanisms have not yet been thoroughly elucidated.

This study employed an ApoE-/- gene knockout mouse model, subjected to a high-fat diet and treated with FMN. We assessed the extent of atherosclerosis damage, the main components within plaques, lipid levels, macrophage M1/M2 phenotype-specific markers, levels of inflammation-related factors, macrophage infiltration, and the molecular markers of the α7nAChR/JAK2/STAT3 axis. The aim was to explore the mechanism of action of FMN in combating atherosclerosis and its potential applications in atherosclerosis treatment. Additionally, this study emphasizes the potential medicinal value of natural compounds, laying a solid theoretical foundation for the use of FMN in the prevention and treatment of atherosclerosis.

## Materials and methods

### Reagents and animals

FMN (HY-N0183), simvastatin (Shanghai Yuanye Bio-Technology Co., Ltd), TRIzol Reagent (CW0580S, CWBIO), miRNA extraction kit (CW0627S, CWBIO), ultrapure RNA extraction kit (CW0581M, CWBIO), ECL ultrasensitive luminescent liquid (Thermo Fisher), BCA protein quantification kit (Elabscience), PVDF membrane (IPVH00010, Millipore), rabbit anti-iNOS (18,985–1-AP, Proteintech), rabbit anti-CD206 (DF4149, Affinity), rabbit anti-CD68 (DF7518, Affinity), rabbit anti-α7nAchR (bs-1049R, Bioss), rabbit anti-p-JAK2 (AF3022, Affinity), rabbit anti-p-STAT3 (AF3293, Affinity), OCT embedding agent (4583, SAKURA), saturated Oil Red O staining solution (G1260, Solarbio), Masson's trichrome staining solution (G1006, Servicebio), and hematoxylin staining solution (ZLI-9610, ZSGB-Bio) were used.

C57/6 J mice (male, 4 weeks old, License Number: SCXK (Su) 2018–0008, Jiangsu Ji cui Yao kang) and ApoE-/- mice (male, 4 weeks old, License Number: SCXK (Su) 2018–0008, Jiangsu Ji cui Yao kang) were housed in a specific pathogen-free (SPF) environment with a temperature range of 20–26 °C and humidity maintained between 40 and 70%. The living environment for all mice was as follows: the temperature was 20–26 °C, humidity was 40%-70%, and free access to food and drinking water was provided. An adaptive feeding period of 7 days was observed. All experiments were approved by the Institutional Animal Care and Use Committee of Traditional Chinese Medicine (Guiyang, China). All mice were anesthetized to death with pentobarbital sodium.

### Inducing cerebral ischemia in mice and experimental group interventions

C57/6 J mice were housed in a specific pathogen-free (SPF) en vironment with a temperature range of 20–26 °C and humidity maintained between 40 and 70%. They had free access to food and water. After 7 days of acclimatization, the animals were divided into experimental groups.

C57/6 J mice were allocated to the blank control group, while ApoE-/- mice were randomly assigned to the model group, model + positive drug group, model + low-dose FMN group, model + medium-dose FMN group, and model + high-dose FMN group. C57/6 J mice were fed normally, whereas ApoE-/- mice were fed a high-fat diet. In the positive control group, after the acclimatization period, simvastatin solution was administered by gavage at a daily dose of 5 mg/kg. In the high-dose FMN group, after the acclimatization period, FMN solution was administered by gavage at a daily dose of 60 mg/kg. In the medium-dose FMN group, after the acclimatization period, FMN solution was administered by gavage at a daily dose of 30 mg/kg. In the low-dose FMN group, after the acclimatization period, FMN solution was administered by gavage at a daily dose of 15 mg/kg. Drug intervention continued for 8 weeks, with daily administrations. Eight weeks later, mice were anesthetized by intraperitoneal injection of sodium pentobarbital (60 mg/kg), and the tissue samples were quickly moved and stored at -80℃. All animal experiments complied with the Guide for the Care and Use of Laboratory Animals (National Institutes of Health Publication No. 85 − 23, revised in 1996) and were reported following the Animal Research Report of In Vivo Experiments (ARRIVE) guidelines.

### Hematoxylin–eosin staining

Following paraffin embedding, sectioning, deparaffinization, and hydration, carotid artery tissue paraffin sections were subjected to staining with hematoxylin for 3–5 min. After rinsing with running water, differentiation was achieved using 1% hydrochloric acid alcohol. Subsequently, a bluing solution was used for counterstaining, followed by eosin staining for 3–5 min. The sections were then dehydrated, coverslipped, and observed under a microscope (BX43, Olympus). Lesion area were measured using Image J software.

### Masson's trichrome staining

Tissue samples were processed as follows: rinsed in running water, dehydrated in a graded ethanol series (70%, 80%, 90%), and cleared in a mixture of absolute alcohol and xylene. After further xylene treatments and immersion in xylene-paraffin mixtures, the samples were embedded in paraffin and sectioned. The sections underwent deparaffinization, standard staining (hematoxylin, Masson's blue, acid hematoxylin, and aniline blue), and dehydration in ethanol. Finally, they were mounted with high-quality medium for microscopic observation (BX43, OLYMPUS). Collagenvolume fraction were measured using Image J software.

### Oil red O staining

The target tissue was surgically prepared and mounted on a sample holder with OCT embedding medium. After freezing until OCT solidified, the tissue sections were cut and thawed at room temperature. Sections were rinsed, fixed in 4% paraformaldehyde, and washed to remove OCT. Oil red O staining was performed for 10 min. Quick differentiation in 60% isopropanol and counterstaining with hematoxylin. Sections were observed under a microscope (CX43, OLYMPUS). Plaque area were measured using Image J software.

### Immunohistochemistry staining

Immunohistochemistry was employed to assess the expression levels of M1/M2 macrophage-specific markers (iNOS, CD206, CD68) in aortic tissue. Sections of mouse aortic plaque tissue were processed through baking, deparaffinization, and hydration, followed by antigen retrieval using citrate buffer. After blocking with 5% BSA to prevent nonspecific binding, the sections were incubated with NF-κB p65 primary antibody (1:100) overnight at 4 °C. Subsequently, they were incubated with goat anti-rabbit secondary antibody (1:100) labeled with horseradish peroxidase. DAB staining was performed, and counterstaining was performed with hematoxylin. After dehydration and clarification, the sections were mounted, and observations were made using a microscope (CX43, OLYMPUS). Protrin relative expression were measured using Image J software.

### Quantitative real-time PCR

Total RNA from carotid artery tissues/cells was extracted using TRIzol reagent, and mRNA/miRNA was extracted using an RNA ultra-pure extraction kit/miRNA ultra-pure extraction kit. The concentration and purity of mRNA were determined using a UV‒Vis spectrophotometer (OD260/OD280). cDNA was synthesized using a reverse transcription kit for RNA/miRNA. Fluorescent quantitative PCR was performed using a fluorescent PCR instrument. The reaction steps were as follows: predenaturation at 95 °C for 10 min, denaturation at 95 °C for 10 s, annealing at 58 °C for 30 s, and extension at 72 °C for 30 s for 40 cycles. β-actin was used as an internal reference, and the relative gene expression was calculated using the 2^-ΔΔCt method. The primer sequences are shown in Table 1.

**Table 1:** Quantitative real-time PCR Primer sequences

Primer Name (Mouse)	Primer Sequence F (5’-3’)	Primer Sequence R (5’-3’)
β-actin	AGGGAAATCGTGCGTGAC	CATACCCAAGAAGGAAGGCT
iNOS	CGTTCCTGGAGGTGCTTGAA	TGGAAGCCACTGACACTTCG
COX-2	CTGGGCCATGGAGTGGACTT	CACTCTGTTGTGCTCCCGAA
IL-1β	GAAATGCCACCTTTTGACAGTG	TGGATGCTCTCATCAGGACAG
IL-6	TCCGGAGAGGAGACTTCACA	TTGCCATTGCACAACTCTTTTC
miR-155-5p	GCGCGTTAATGCTAATTGTGAT	AGTGCAGGGTCCGAGGTATT
IL-10	GTCATCGATTTCTTCCCTGTG	ACTCATGGCTTTGTAGATGCCT
Arg-1	TTGGGTGGATGCTCACACTG	GTACACGATGTCTTTGGCAGA
SHIP1	TGAGGGAGAAGCTCTATGACTTT	GAAGGCTCCCATTGCCTCATAG

### Western blot detection

A certain amount of carotid artery tissue was taken, added to RIPA lysis buffer, and ground using a tissue grinder to extract total tissue protein (for cells: collect the cells, discard the culture medium, and extract total protein using RIPA lysis buffer). Centrifuge at 12,000 rpm at 4 °C for 10 min, collect the supernatant, and quantitate the total protein using a BCA protein quantification kit. After denaturation of the protein samples, sodium dodecyl sulfate‒polyacrylamide gel electrophoresis (SDS‒PAGE) was performed for 1.5 h, followed by electroblotting at a constant current of 300 mA for 1 h. The PVDF membrane (Millipore) was blocked with skim milk powder, and the primary antibody was incubated overnight at 4 °C. The next day, the PVDF membrane was incubated with the secondary antibody at room temperature for 2 h, immersed in a chemiluminescent reagent, and placed in an ultrasensitive chemiluminescent imaging system for visualization. The blots were cut prior to hybridization with antibodies, so there are no images showing full length membranes.

### Biochemical analysis

Serum samples were collected and centrifuged at 1000 × g for 20 min, and the levels of TC (total cholesterol), TG (triglycerides), LDL-C (low-density lipoprotein cholesterol), HDL-C (high-density lipoprotein cholesterol), and ox-LDL were measured using biochemical assay kits according to the kit instructions. The absorbance (OD) values for each well were determined at the respective wavelengths using an enzyme-linked immunosorbent assay (ELISA) reader.

### Statistical analysis

Statistical analysis and graphing were performed using GraphPad Prism 8.0.1 software. All experiments were repeated three times, and quantitative results are expressed as the mean ± standard deviation (X ± S). One-way analysis of variance (ANOVA) was used for quantitative comparisons among multiple groups, with a significance level set at α = 0.05. A *p* value of less than 0.05 was considered statistically significant.

## Results

### Histopathological changes in the carotid artery tissues of each group were observed through H&E, Masson, and Oil Red O staining

**Fig. 1:**
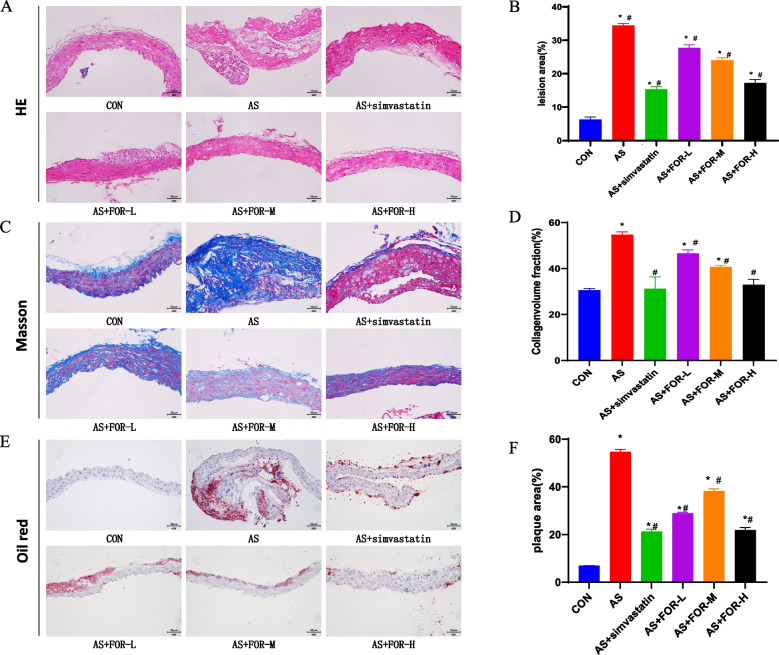
Observation of histopathological changes in carotid artery tissues in all groups using HE, Masson, and Oil Red O staining. **A**, **C**, **E** Representative morphological images of H&E,Masson and Oil Red O staining (Magnification, × 40;Scale bar = 50 μm); **B**, **D**, **F** Quantitative examination of lesion area,collagenvolume fraction and plaque area (The data are presented as the mean ± SD, *n* = 3–6; ^*^ indicates *P* < 0.05 compared to the CON group, ^#^ indicates *P* < 0.05 compared to the AS group)

As shown in Fig. 1, through HE staining, the morphological changes in aortic tissues of the various groups of mice were examined. In the control group, the aortic intimal structure remained intact, showing no signs of proliferation or luminal narrowing. In contrast, the AS model group displayed a significantly thickened aortic intima with the formation of atherosclerotic plaques, and these plaques exhibited a substantial area. Comparatively, the degree of intimal proliferation in the mice was inversely correlated with the dosage of FMN, with a notable reduction in plaque size and luminal stenosis as the dosage of FMN increased, particularly notable in the high-dosage group.

Masson's staining was employed to assess the fibrosis status of aortic plaques in all groups of mice. In the control group, the arterial intimal structure remained intact, and only minimal fibrosis was observed. Conversely, the model group exhibited a conspicuously thickened aortic intima with fibrous caps on the plaques, beneath which necrotic debris, foam cells, and inflammatory cells were eviden. Similarly, relative to the AS model group, FMN administration at varying dosages ameliorated the fibrosis status of aortic plaques in mice, with the middle-dosage group demonstrating the best improvement.

Quantification of AS plaque formation was conducted by Oil Red O staining at the root of the carotid artery to assess lipid deposition. In the control group, no intimal thickening was observed, and the number of stained plaques was minimal. In contrast, the AS model group displayed intimal thickening and a significant increase in the area of red-stained plaques compared to the control group. Furthermore, relative to the model group, FMN at the three administered dosages exhibited favorable effects on plaque formation.

### Biochemical analysis of lipid content changes in mice serum in different groups

**Fig. 2:**
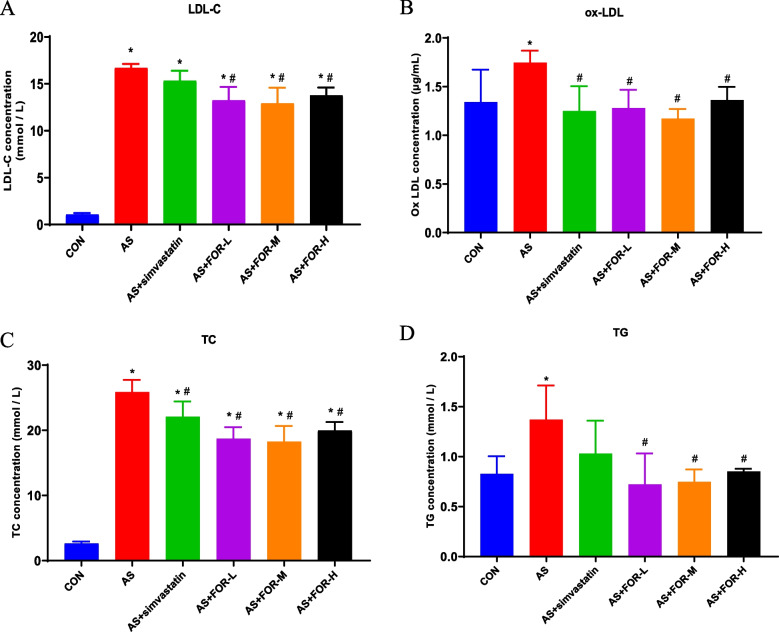
Changes in Lipid Content in Mouse Serum in Different Groups as Analyzed Biochemically (The data are presented as the mean ± SD, *n* = 3–6. ^*^ indicates *P* < 0.05 compared to the CON group, ^#^ indicates *P* < 0.05 compared to the AS group)

As shown in Fig. 2, compared to the normal control group, the AS model group of mice exhibited a significant increase in the levels of LDL-C, ox-LDL, TC, and TG in serum; When compared to the AS group, the low-, medium-, and high-dose FMN treatment groups of mice displayed significant decreases in serum levels of LDL-C, ox-LDL, TC, and TG; In comparison to the AS group, the positive drug group treated with simvastatin showed significant reductions in serum levels of ox-LDL,TC, and LDL-C, while TG levels showing a decrease without significant differences.

Immunohistochemical staining was used to observe the changes in the expression of CD68, CD206, and iNOS in carotid artery tissues of all groups.

**Fig. 3:**
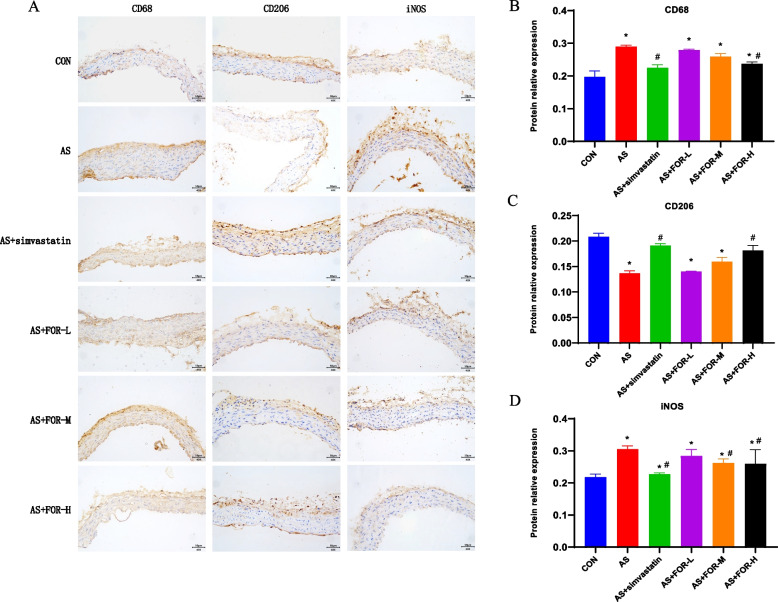
Immunohistochemical Staining to Observe the Expression Changes of CD68, CD206, and iNOS in Carotid Artery Tissues in Various Groups. **A** Representative morphological images of immunohistochemical staining(Magnification, × 40;Scale bar = 50 μm); **B**-**D** The changes of protein expression levels in CD68,CD206 and iNOS for each group (The data are presented as the mean ± SD, *n* = 3–6; ^*^ indicates *P* < 0.05 compared to the CON group, ^#^ indicates *P* < 0.05 compared to the AS group)

As shown in Fig. 3, compared to the normal control group, the expression levels of CD68 and iNOS in the carotid artery tissues of the AS model group increased, while CD206 expression decreased; Compared to the AS model group, the groups treated with the positive drugs simvastatin and FMN at low-, medium-, and high-doses showed varying degrees of decrease in CD68 and iNOS expression and an increase in CD206 expression in carotid artery tissues,with significant changes observed in the high-dose group.

### Quantitative real-time PCR was used to assess the changes in mRNA and miR-155-5p expression in carotid artery tissues among the different groups

**Fig. 4:**
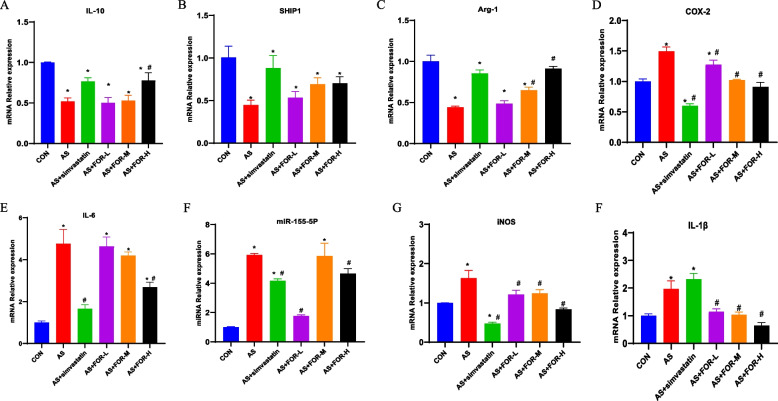
RTq-PCR Analysis of mRNA and miR-155-5p Expression Changes in Cervical Aortic Tissues in Various Groups (The data are presented as the mean ± SD, *n* = 3–6. ^*^ indicates *P* < 0.05 compared to the CON group, ^#^ indicates *P* < 0.05 compared to the AS group)

As shown in Fig. 4, compared to the normal control group, the expression of iNOS, COX-2, IL-1β, IL-6, and miR-155-5p mRNA levels in the cervical aortic tissues of the AS model group were significantly increased. In contrast, the expression of IL-10, SHIP1, and Arg-1 mRNA levels significantly decreased; Compared to the AS model group, the simvastatin group showed significant reductions in the expression levels of iNOS, COX-2, IL-6, and miR-155-5p mRNA in the cervical aortic tissues, and there was also a decrease in IL-1β mRNA, although not statistically significant.While the expression of IL-10, SHIP1, and Arg-1 mRNA significantly increased; The low-dose FMN group exhibited significant reductions in iNOS, COX-2, and miR-155-5p mRNA expression in cervical aortic tissues, with decreases in IL-1β and IL-6 mRNA levels but no significant differences when compared to the AS model group,while the expression of IL-10, SHIP1, and Arg-1 mRNA increased, but without significance; The medium-dose FMN group displayed significant reductions in the expression of iNOS, COX-2, IL-1β, and IL-6 mRNA levels in cervical aortic tissues, with no significant changes in miR-155-5p mRNA expression when compared to the AS model, while the expression of IL-10, SHIP1, and Arg-1 mRNA levels all increased, but only the change in Arg-1 mRNA expression was significant; Compared to the AS model group,the high-dose FMN group showed significant reductions in iNOS, IL-1β, IL-6, and miR-155-5p mRNA expression in cervical aortic tissues.Notably, the expression levels of IL-10, SHIP1, and Arg-1 mRNA expression all significantly increased.

### Western blot analysis of protein expression changes in cervical aortic tissues in various groups

**Fig. 5:**
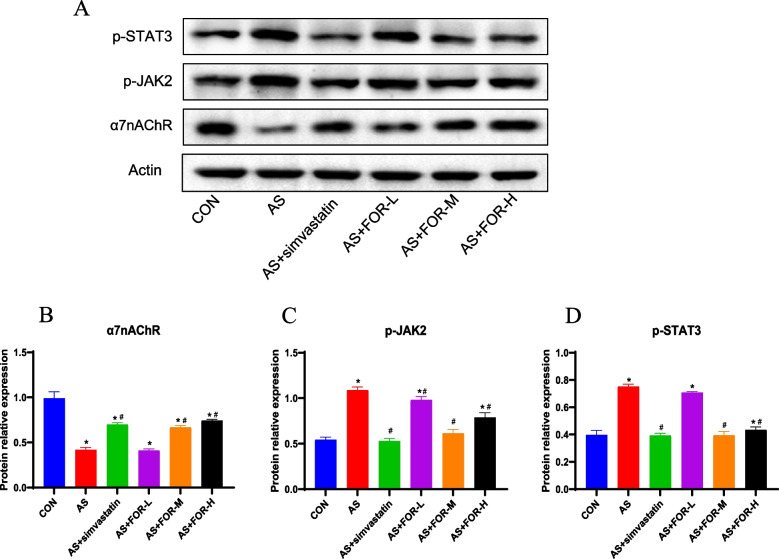
Western Blot Analysis of Protein Expression Changes in Cervical Aortic Tissues in Different Groups (The data are presented as the mean ± SD, *n* = 3–6. ^*^ indicates *P* < 0.05 compared to the CON group, ^#^ indicates *P* < 0.05 compared to the AS group)

As shown in Fig. 5, compared to the normal control group, the AS model group exhibited a significant decrease in α7nAChR protein expression in cervical aortic tissues, along with significant increases in p-JAK2 and p-STAT3 protein expression; In comparison to the AS model group, the positive control group treated with simvastatin exhibited a significant increase in α7nAChR protein expression and significant decreases in p-JAK2 and p-STAT3 protein expression in cervical aortic tissues; Compared to the AS model group, the low-dose FMN group displayed a significant decrease in p-JAK2 protein expression, with no significant differences in α7nAChR and p-STAT3 protein expression; In contrast, the medium-dose FMN group exhibited a significant increase in α7nAChR protein expression, along with significant decreases in p-JAK2 and p-STAT3 protein expression when compared to the AS model group; Moreover, the high-dose FMN group demonstrated a significant increase in α7nAChR protein expression and significant.

## Discussion

In recent years, extensive research has underscored inflammation as a central pathological mechanism in AS, closely intertwined with its development and prognosis [20]. During the course of AS progression, the differentiation and phenotypic transformation of macrophages play a pivotal role in shaping the inflammatory microenvironment within arterial walls [21]. Antiplatelet medications and statins currently stand as the most commonly employed pharmaceutical agents in the clinical management of atherosclerotic cardiovascular disease (ASCVD) [22]. However, the high cost and side effects associated with long-term medications currently available in the market and those in synthetic drug development pipelines underscore the urgent need for viable alternatives. Traditional Chinese medicine (TCM) plays a crucial role in the prevention and treatment of cardiovascular diseases and is characterized by its multicomponent and multitarget therapeutic approach that can act simultaneously or synergistically to address disease pathology. FMN, a natural flavonoid component, held a focal position in our research. We employed an ApoE-/- gene knockout mouse model and subjected them to a high-fat diet, followed by FMN intervention, to comprehensively investigate the mechanistic role of FMN in countering atherosclerosis and its potential applications in the therapeutic management of atherosclerosis.

In this study, various doses of FMN were administered to assess their impact on aortic morphology. The results revealed a significant reduction in atherosclerotic fibrous cap prominence, lipid deposition, plaque area, and structural alterations on the surface of the carotid artery in ApoE-/- mice following FMN intervention. Furthermore, the effectiveness of FMN became more pronounced with increasing dosage. Concurrently, we assessed changes in serum lipid content in mice. Notably, the FMN intervention led to a significant reduction in LDL-C, ox-LDL, TC, and TG levels in the low-, medium-, and high-dose groups. These experimental findings provide compelling evidence for the anti-inflammatory and atherosclerosis-mitigating properties of FMN.

In the context of atherosclerosis, macrophage polarization plays a crucial role [23]. CD68 and iNOS are established markers for M1-type macrophages, while CD206 is one of the markers for M2-type macrophages [24]. We employed immunohistochemical staining to observe the changes in the expression of CD68, CD206, and iNOS in the carotid artery tissues of different treatment groups. The results demonstrated that, in comparison to the AS model group, the low-, medium-, and high-dose FMN treatment groups exhibited varying degrees of reduced CD68 and iNOS expression, along with increased CD206 expression. These findings align with the trends observed in numerous previous studies [25–28], indicating a decrease in M1-type macrophages and an increase in M2-type macrophages. Subsequently, we conducted PCR analysis of relevant cytokines. The results further demonstrated that FMN intervention could inhibit the expression of iNOS, COX-2, miR-155-5p, IL-6, and IL-1β mRNA, while promoting the expression of IL-10, SHIP1, and Arg-1 mRNA. Previous experimental studies have confirmed that iNOS levels are elevated in atherosclerosis models compared to control groups [29]. As our results showed, the protein and mRNA levels of iNOS increased in the model group, while FMN intervention significantly reduced iNOS levels. Additionally, a high-fat diet induces the production of IL-1β, whereas FMN reduces IL-1β production. Research indicates that iNOS serves as a crucial marker for M1-type macrophages. Furthermore, IL-1β represents an inflammatory cytokine secreted by M1 macrophages and is involved in the progression of atherosclerosis [30, 31]. As atherosclerosis develops, the number of M2 macrophages decreased, and they express cytokines such as Interleukin (IL) -10, which have been identified as anti-inflammatory and anti-atherosclerotic [23]. In summary, our research results suggest that FMN can inhibit macrophage polarization toward the M1 phenotype (iNOS), suppress the release of proinflammatory factors, such as IL-1β, and promote M2-type polarization, thereby reducing inflammation and alleviating atherosclerosis.

When considering pathways related to inflammation in atherosclerosis (AS), it becomes apparent that their complexity is evident, including the Janus kinase/signal transducer and activator of transcription (JAK/STAT) signaling pathway. Extensive research has clarified the critical role of JAK/STAT signaling pathway activation in regulating atherosclerosis-related inflammation [32, 33]. In the AS model group, proteins associated with the JAK/STAT signaling pathway were found to be activated [34], and our study's results are consistent with this observation. Moreover, FMN intervention, whether at low-, medium-, or high-doses, effectively inhibited the expression of p-JAK2 and p-STAT3. Tang's research suggests that inhibiting JAK/STAT signal transduction can alleviate atherosclerosis in ApoE-/- mice [35]. The results from a rabbit model of atherosclerosis similarly indicate that inhibiting JAK/STAT signal transduction can reduce atherosclerosis in ApoE-/- mice [36]. Our results show that FMN can also modulate atherosclerotic inflammation by suppressing the JAK/STAT signaling pathway. In addition to studying the JAK/STAT signaling pathway, we assessed the protein expression of the critical effector molecule α7nAChR in the cholinergic anti-inflammatory pathway. Research suggests that α7nAChR plays a crucial regulatory role in atherosclerosis [37]. However, in contrast to the suppression of p-JAK2 and p-STAT3 protein expression, different doses of FMN significantly increased the expression of α7nAChR compared to the AS model group. The results from Ulleryd's study demonstrate that stimulating α7 nicotinic acetylcholine receptors (α7nAChR) can inhibit atherosclerosis by regulating bone marrow cell immune responses [38], and certain traditional Chinese medicines or natural components can also activate α7nAchR signal transduction, exerting anti-inflammatory effects [39, 40]. Therefore, based on our experimental results, FMN can improve atherosclerotic inflammation by activating the expression of α7nAchRs.

## Conclusion

The research findings indicate that FMN can modulate macrophage polarization, inhibit the JAK/STAT signaling pathway, and promote the expression of α7nAChR, thereby reducing inflammation and ameliorating atherosclerosis. Nevertheless, the precise mechanisms through which FMN stabilizes atherosclerosis-related pathways by regulating macrophage polarization, particularly within the context of cholinergic anti-inflammatory pathways centered on α7nAChR, require further investigation.

### Supplementary Information

**Supplementary material 1.**

**Supplementary material 2.**

## Data Availability

The data that support the fndings of this study are available on request from the corresponding author (Qiansong He), uponreasonable request.
