# Supplementary material for: Elucidating the mechanisms of formononetin in modulating atherosclerotic plaque formation in ApoE-/- mice
Source: BMC Cardiovasc Disord. 2024 Feb 22;24:121. doi: 10.1186/s12872-024-03774-6 (PMC10882812; doi:10.1186/s12872-024-03774-6)
Supplement: Supplementary file 1 — Supplementary material 1. [file 12872_2024_3774_MOESM1_ESM.docx]

**Original images of Figure 5A**

p-STAT3 (86KDa)


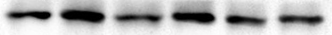


**
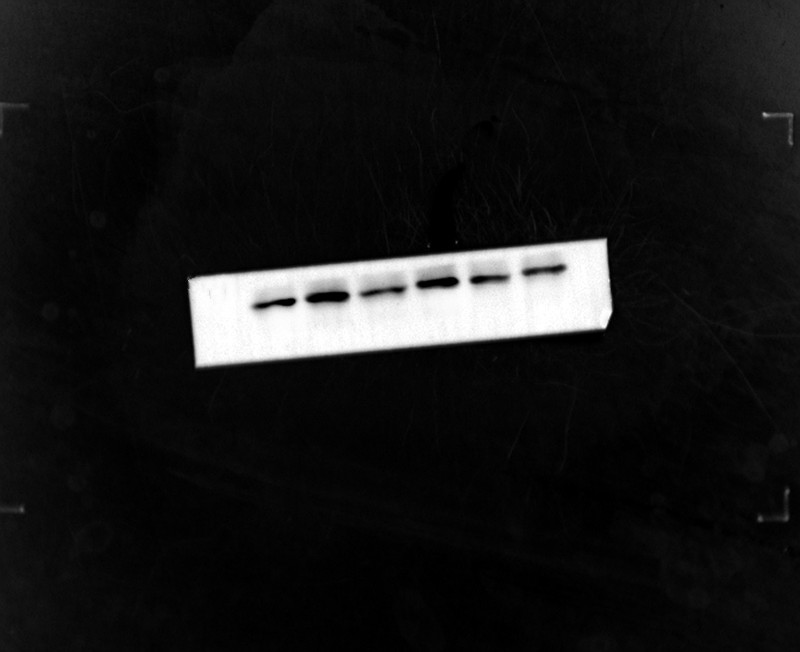
**

AS

AS+simvastatin

AS+FOR-H

AS+FOR-LM

AS+FOR-L

CON

p-JAK2 (120KDa)

**
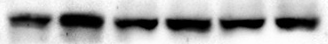
**

**
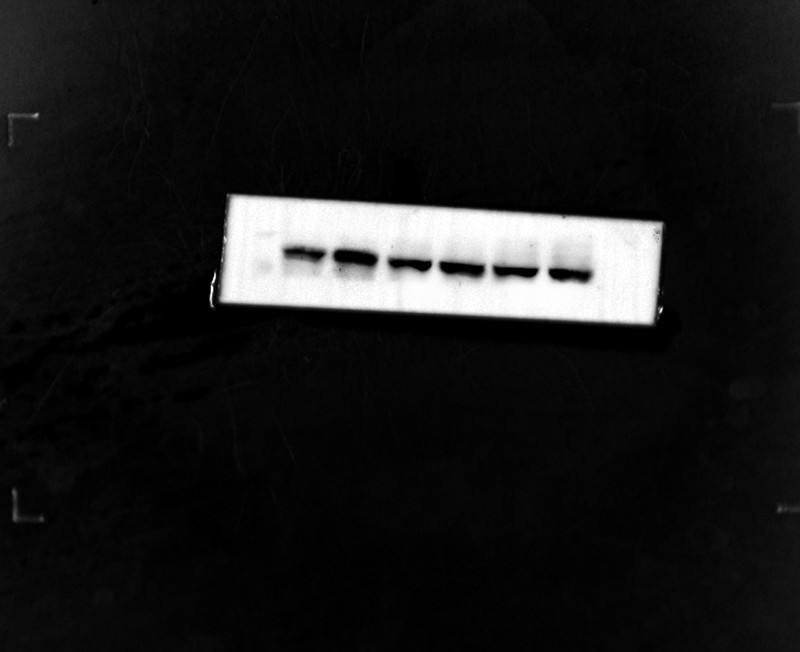
**

AS

AS+simvastatin

AS+FOR-H

AS+FOR-M

AS+FOR-L

CON

α7nAchR (55KDa)

**
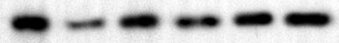
**

**
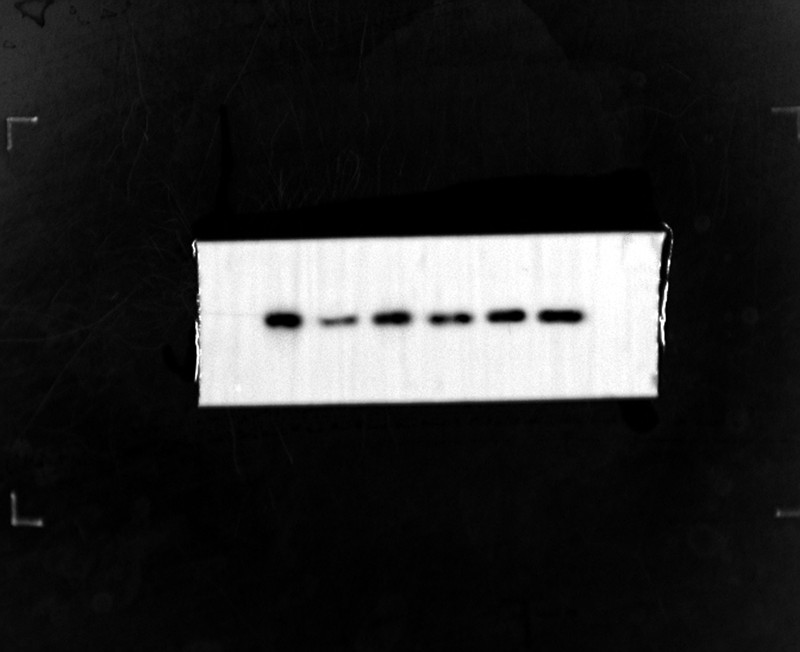
**

AS+FOR-H

AS+FOR-M

AS+simvastatin

AS+FOR-L

CON

AS

Actin (42KDa)

**
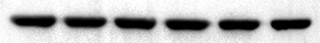
**

**
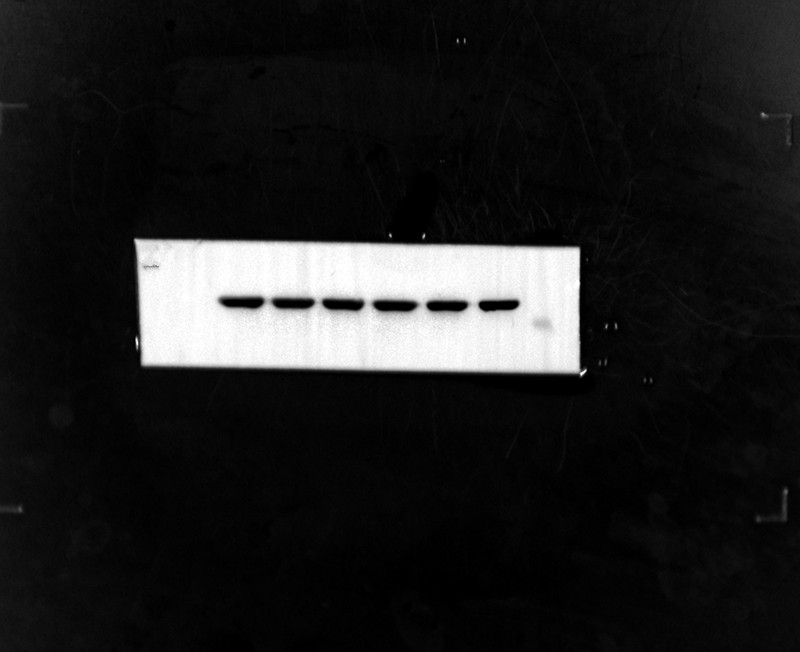
**

CON

AS+FOR-H

AS+FOR-M

AS+FOR-L

AS+simvastatin

AS
