# Supplementary figures and images for: Elucidating the mechanisms of formononetin in modulating atherosclerotic plaque formation in ApoE-/- mice
Source: BMC Cardiovasc Disord. 2024 Feb 22;24:121. doi: 10.1186/s12872-024-03774-6 (PMC10882812; doi:10.1186/s12872-024-03774-6)

## Slide 1
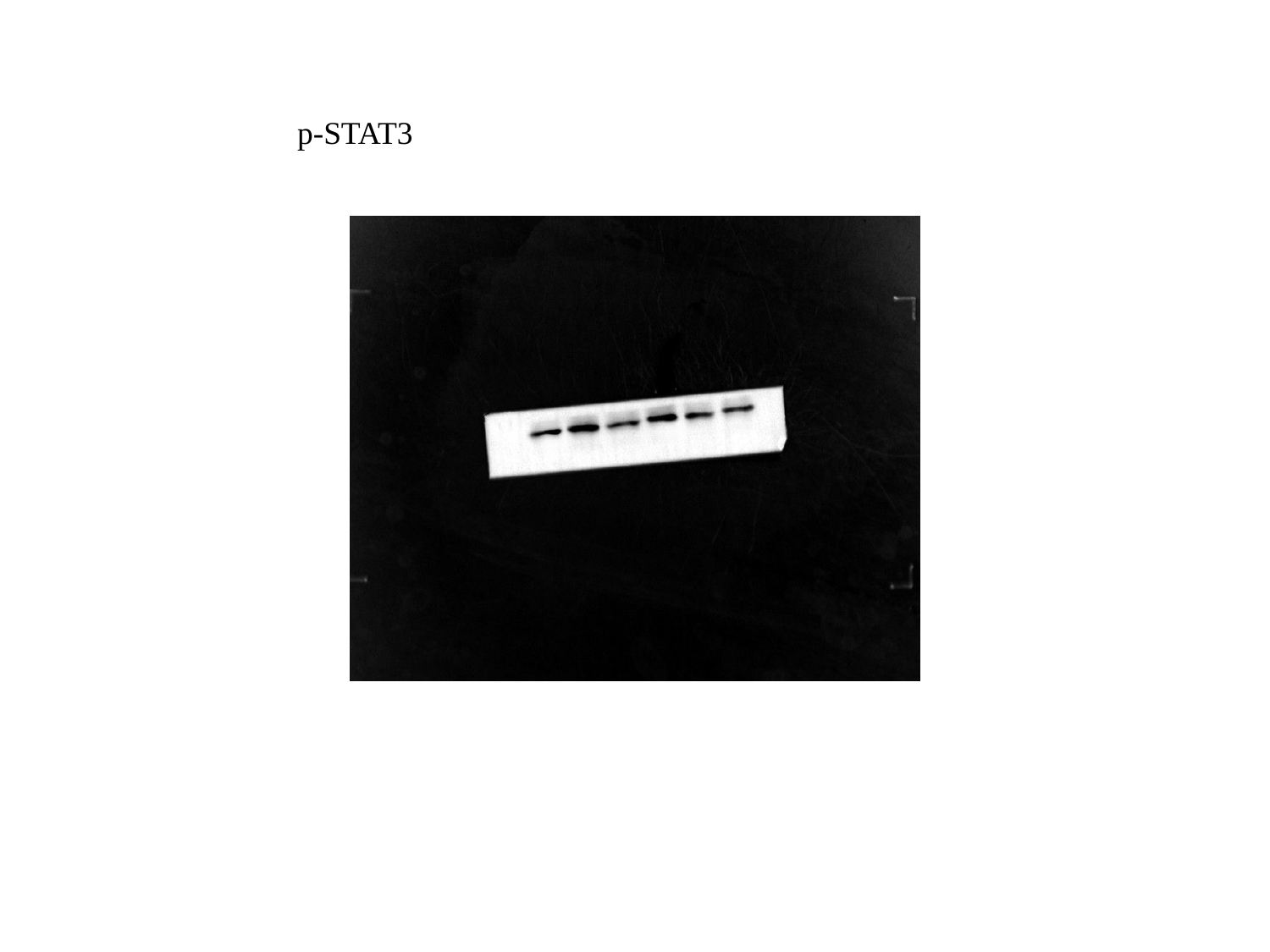

p-STAT3

## Slide 2
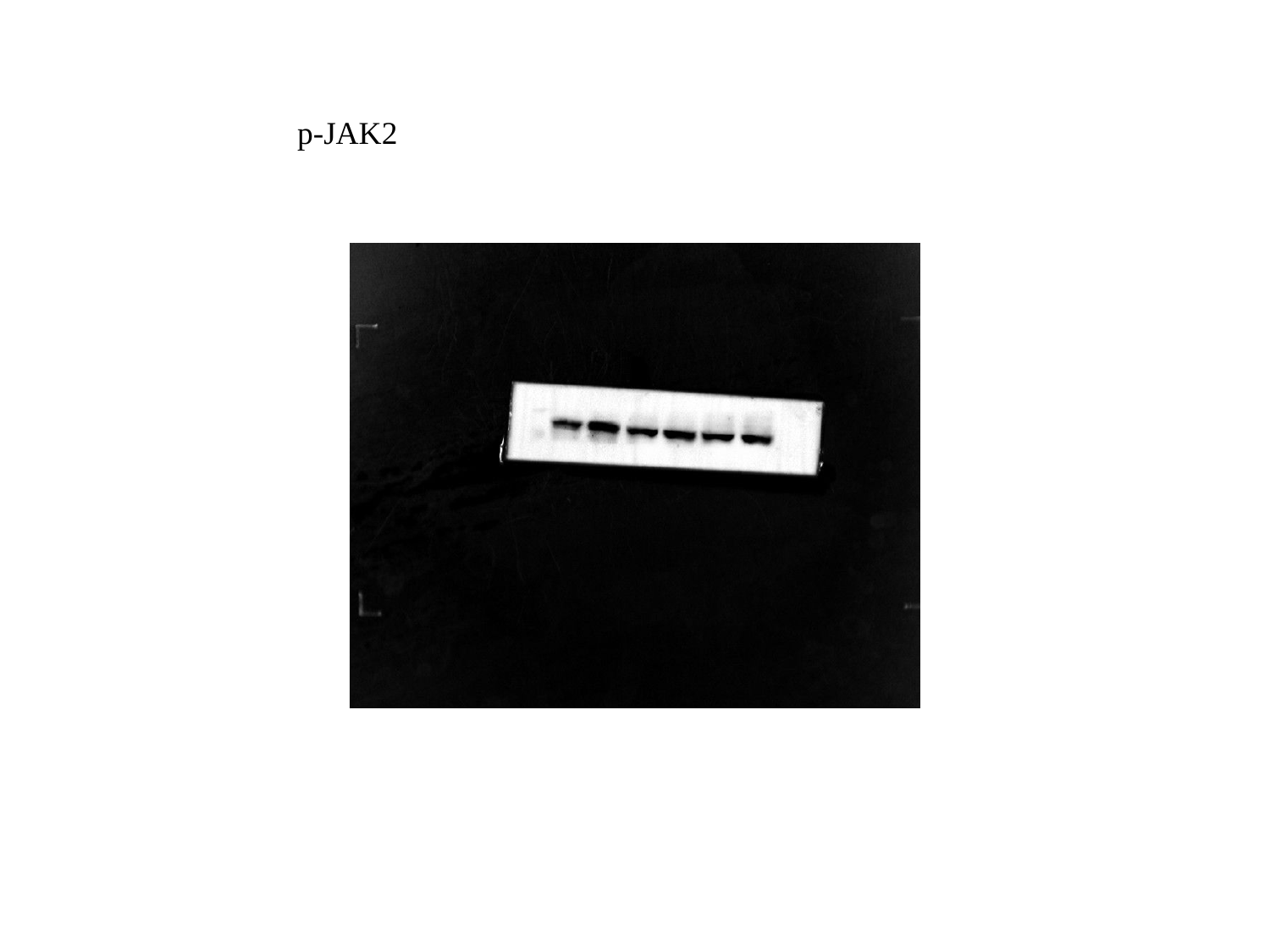

p-JAK2

## Slide 3
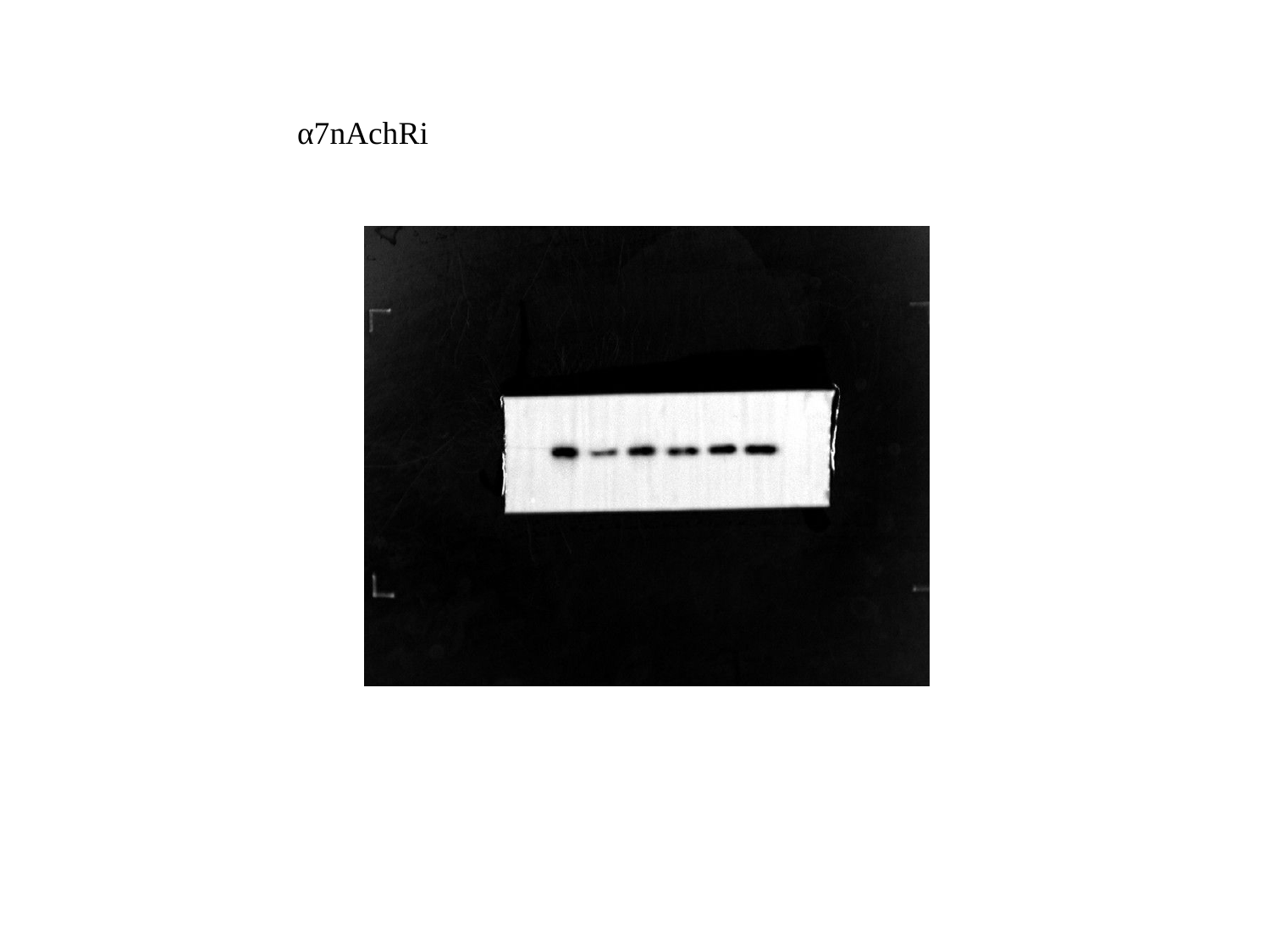

α7nAchRi

## Slide 4
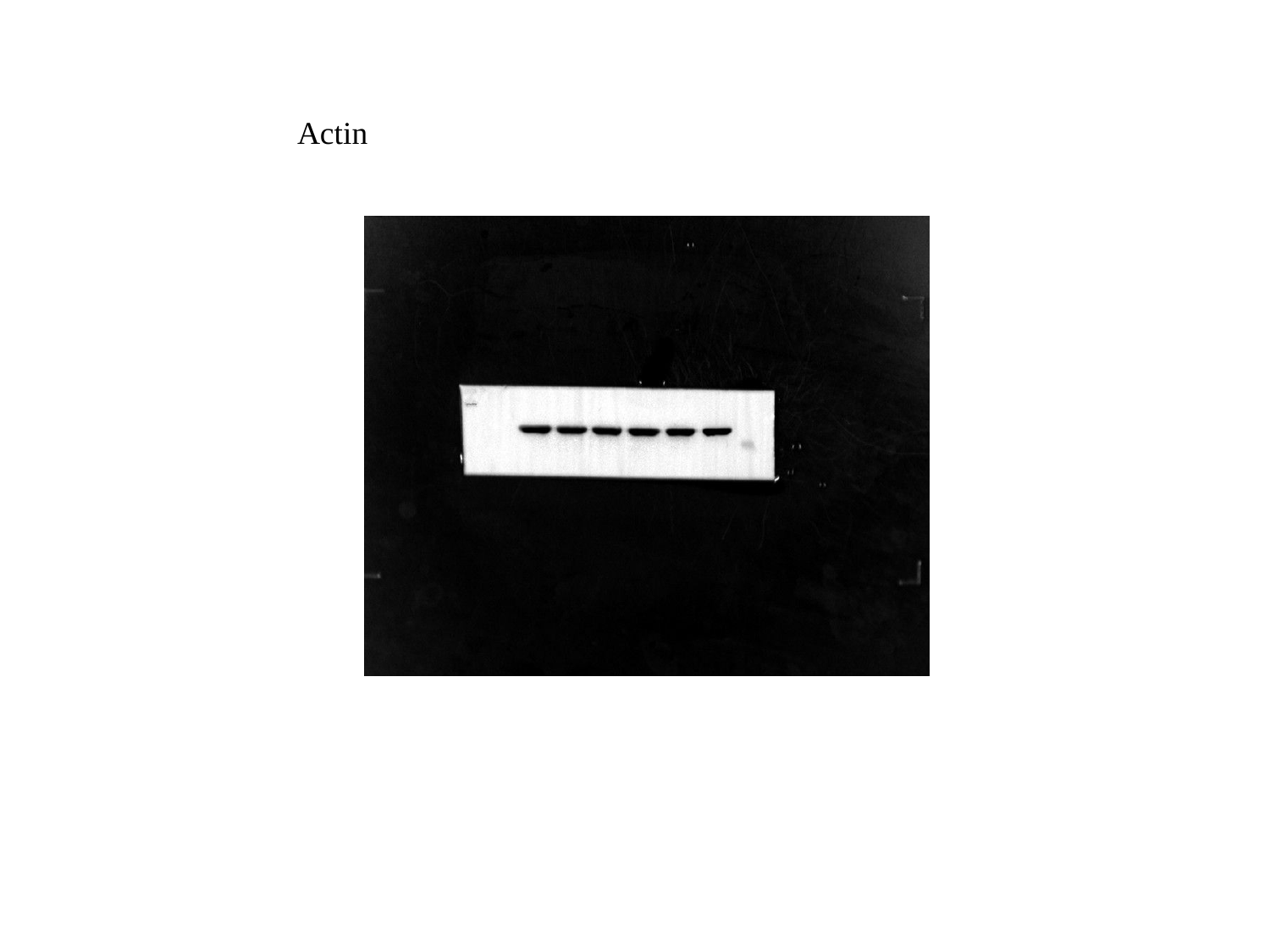

Actin

Supplement: Supplementary file 2 — Supplementary material 2. [file 12872_2024_3774_MOESM2_ESM.ppt]
